# Supplementary material for: Increased academic stress is associated with decreased plasma BDNF in Chilean college students
Source: PeerJ. 2023 Nov 3;11:e16357. doi: 10.7717/peerj.16357 (PMC10629390; doi:10.7717/peerj.16357)
Supplement: Supplemental Information 2 [file peerj-11-16357-s002.pdf]

Variable	Descripción
.ID	ID participantes
-9	Valores perdidos
.Sexo	Sexo: 0=F;1=M
	Beck-II
BB.FeNac	Fecha nacimiento
BB.FeEv	Fecha Evaluación Beck-II basal
BB.Edad	Edad evaluación Beck-II basal
BB.Car	Carrera evaluación Beck-II basal
BB.Sem	Semestre evaluación Beck-II basal
BB.1	Item 1: Tristeza
BB.2	Item 2: Pesimismo
BB.3	Item 3: Sentimientos de Fracaso
BB.4	Item 4: Pérdida de placer
BB.5	Item 5: Sentimientos de culpa
BB.6	Item 6: Sentimientos de castigo
BB.7	Item 7: Insafisfaccion con uno mismo
BB.8	Item 8: Autocrtícas
BB.9	Item 9: Pensamos o deseos de suicidio
BB.10	Item 10: Llanto
BB.11	Item 11: Agitación
BB.12	Item 12: Pérdida de interés
BB.13	Item 13: Indecision
BB.14	Item 14: Inutilidad
BB.15	Item 15: Pérdida de Energía
BB.16	Item 16: Cambio Patrón Sueño
BB.17	Item 17: Irritabilidad
BB.18	Item 18: Cambio en el apetito
BB.19	Item 19: Dificultad de Concentración
BB.20	Item 20:Cansancio o fatiga
BB.21	Item 21: Pérdida de inetrés por el sexo
BB.PT	Puntaje total Beck-II basal

Variable	Descripción
FB.FeNac	Fecha nacimiento
FB.FeEv	Fecha Evaluación Beck-II final
FB.Edad	Edad evaluación Beck-II final
FB.Car	Carrera evaluación Beck-II final
FB.Sem	Semestre evaluación Beck-II final
FB.1	Item 1: Tristeza
FB.2	Item 2: Pesimismo
FB.3	Item 3: Sentimientos de Fracaso
FB.4	Item 4: Pérdida de placer
FB.5	Item 5: Sentimientos de culpa
FB.6	Item 6: Sentimientos de castigo
FB.97	Item 7: Insafisfaccion con uno mismo
FB.8	Item 8: Autocrtícas
FB.9	Item 9: Pensamos o deseos de suicidio
FB.10	Item 10: Llanto
FB.11	Item 11: Agitación
FB.12	Item 12: Pérdida de interés
FB.13	Item 13: Indecision
FB.14	Item 14: Inutilidad
FB.15	Item 15: Pérdida de Energía
FB.16	Item 16: Cambio Patrón Sueño
FB.17	Item 17: Irritabilidad
FB.18	Item 18: Cambio en el apetito
FB.19	Item 19: Dificultad de Concentración
FB.20	Item 20:Cansancio o fatiga
FB.21	Item 21: Pérdida de inetrés por el sexo
FB.PT	Puntaje total Beck-II final
	ISCO-II academic stress inventory

Variable	Descripción
BS.FeNac	Fecha nacimiento
BS.FeEv	Fecha Evaluación SISCO-II basal
BS.Edad	Edad evaluación SISCO-II basal
BS.Car	Carrera evaluación SISCO-II basal
BS.Sem	Semestre evaluación SISCO-II basal
BS.P1	1:Sí 0:No
BS.P2	Número
BS.P3_1	Sobrecarga de tareas y trabajos académicos
BS.P3_2	La personalidad y carácter de los profesores
BS.P3_3	Las evaluaciones de los profesores (exámenes, ensayos, trabajos de investigación, etc.)
BS.P3_4	El tipo de trabajo que te piden los profesores (consulta de mapas, fichas de trabajo, ensayos, mapas conceptuales, etc.)
BS.P3_5	No entender los temas que se abordan en la clase
BS.P3_6	Participación en clase (responder a preguntas, exposiciones, etc.)
BS.P3_7	Tiempo limitado para hacer el trabajo
BS.P3_8	Los compañeros de grupo progresan más rápido en tareas y/o trabajos académicos
BS.P4_1	Trastornos del sueño (insomnio o pesadillas)
BS.P4_2	Fatiga crónica (cansancio permanente)
BS.P4_3	Dolores de cabeza o migrañas
BS.P4_4	Problemas de digestión, dolor abdominal o diarrea
BS.P4_5	Rascarse, morderse las uñas, frotarse, etc.
BS.P4_6	Somnolencia o mayor necesidad de dormir.
BS.P4_7	Dolores musculares y/o contracturas
BS.P4_8	Reacciones cutáneas (sarpullido, descamación, etc.)
BS.P4_9	Inquietud (incapacidad de relajarse y estar tranquilo)
BS.P4_10	Ansiedad, angustia o desesperación
BS.P4_11	Aumento o reducción del consumo de alimentos
BS.P4_12	Sentimientos de depresión y tristeza (decaído)
BS.P4_13	Sentimiento de agresividad o aumento de irritabilidad
BS.P4_14	Cambios bruscos de humor
BS.P4_15	Conflictos o tendencia a polemizar o discutir
BS.P4_16	Aislamiento de los demás
BS.P4_17	Desgano para realizar tus labores de estudiante
BS.P5_1	Elaboración de un plan de ejecución de sus tareas
BS.P5_2	Elogios a sí mismo
BS.P5_3	Ventilación y confidencias (verbalización de la situación que preocupa)
BS.P5_4	Intenté sacar algo positivo o beneficioso de la situación estresante
BS.P5_5	Practicar un pasatiempo (actividad física, leer, ver series, redes sociales, etc.)
BS.P5_6	Acompañarse de un ser querido (familia, mascotas, amigos, etc.)
BS.EstSIS	Total Estresores SISCO-II evaluación basal
BS.RfsSIS	Total Reacciones físicas y psicológicas SISCO-II evaluación basal
BS.RcsSIS	Total reacciones del comportamiento social SISCO-II evaluación basal
BS.RxTSIS	Total Reacción Total SISCO-II evaluación basal
BS.EASIS	Total medida de estrés académico SISCO-II evaluación basal
BS.AfSIS	Total Afrontamiento SISCO-II (Factor 4) evaluación basal
BS.TSIS.Af	Total SISCO-II + afrontamiento evaluación basal

Variable	Descripción
FS.FeNac	Fecha nacimiento
FS.FeEv	Fecha Evaluación SISCO-II final
FS.Edad	Edad evaluación SISCO-II final
FS.Car	Carrera evaluación SISCO-II final
FS.Sem	Semestre evaluación SISCO-II final
FS.P1	1:Sí 0:No
FS.P2	Número
FS.P3_1	Sobrecarga de tareas y trabajos académicos
FS.P3_2	La personalidad y carácter de los profesores
FS.P3_3	Las evaluaciones de los profesores (exámenes, ensayos, trabajos de investigación, etc.)
FS.P3_4	El tipo de trabajo que te piden los profesores (consulta de mapas, fichas de trabajo, ensayos, mapas conceptuales, etc.)
FS.P3_5	No entender los temas que se abordan en la clase
FS.P3_6	Participación en clase (responder a preguntas, exposiciones, etc.)
FS.P3_7	Tiempo limitado para hacer el trabajo
FS.P3_8	Los compañeros de grupo progresan más rápido en tareas y/o trabajos académicos
FS.P4_1	Trastornos del sueño (insomnio o pesadillas)
FS.P4_2	Fatiga crónica (cansancio permanente)
FS.P4_3	Dolores de cabeza o migrañas
FS.P4_4	Problemas de digestión, dolor abdominal o diarrea
FS.P4_5	Rascarse, morderse las uñas, frotarse, etc.
FS.P4_6	Somnolencia o mayor necesidad de dormir.
FS.P4_7	Dolores musculares y/o contracturas
FS.P4_8	Reacciones cutáneas (sarpullido, descamación, etc.)
FS.P4_9	Inquietud (incapacidad de relajarse y estar tranquilo)
FS.P4_10	Ansiedad, angustia o desesperación
FS.P4_11	Aumento o reducción del consumo de alimentos
FS.P4_12	Sentimientos de depresión y tristeza (decaído)
FS.P4_13	Sentimiento de agresividad o aumento de irritabilidad
FS.P4_14	Cambios bruscos de humor
FS.P4_15	Conflictos o tendencia a polemizar o discutir
FS.P4_16	Aislamiento de los demás
FS.P4_17	Desgano para realizar tus labores de estudiante
FS.P5_1	Elaboración de un plan de ejecución de sus tareas
FS.P5_2	Elogios a sí mismo
FS.P5_3	Ventilación y confidencias (verbalización de la situación que preocupa)
FS.P5_4	Intenté sacar algo positivo o beneficioso de la situación estresante
FS.P5_5	Practicar un pasatiempo (actividad física, leer, ver series, redes sociales, etc.)
FS.P5_6	Acompañarse de un ser querido (familia, mascotas, amigos, etc.)
FS.EstSIS	Total Estresores SISCO-II evaluación final
FS.RfsSIS.2	Total Reacciones físicas y psicológicas SISCO-II evaluación final
FS.RcsSIS	Total reacciones del comportamiento social SISCO-II evaluación final
FS.RxTSIS	Total Reacción Total SISCO-II evaluación final
FS.EASIS	Total medida de estrés académico SISCO-II evaluación final
FS.AfSIS	Total Afrontamiento SISCO-II (Factor 4) evaluación basal
FS.TSIS.Af	Total SISCO-II + afrontamiento evaluaciónfinal

Variable	Descripción
	**Basic data**

Variable	Descripción
BDG.FeNac	Fecha Nacimiento
BDG.FeEv	Fecha Evaluación datos generales basal
BDG.Edad	Edad evaluación datos generales basal
BDG.Car	Carrera datos generales basal
BDG.Sem	Semestre datos generales basal
BDG.TTOPsq	T. psiquiátrico datos generales basal
BDG.TTOPsic	Tto psicológico datos generales basal
BDG.TTOfar	Tto fármaco datos generales basal
BDG.TTOfarNom	Si: Cuál(es)
BDG.Fuma	Fuma datos generales basal
BDG.FumaNro	Si: N°
BDG.Bebe	Bebe datos generales basal
BDG.BebeNro	Si: N°
BDG.AcFis	Ac. Física datos generales basal
BDG.AcFisNro	Si: N°
BDG.AcFisCual	Si:
FDG.FeNac	Fecha Nacimiento
FDG.FeEv	Fecha Evaluación datos generales final
FDG.Edad	Edad evaluación datos generales final
FDG.Car	Carrera datos generales final
FDG.Sem	Semestre datos generales final
FDG.TTOPsq	T. psiquiátrico datos generales final
FDG.TTOPsic	Tto psicológico datos generales final
FDG.TTOfar	Tto fármaco datos generales final
FDG.TTOfarNom	Si: Cuál(es)
FDG.Fuma	Fuma datos generales final
FDG.FumaNro	Si: N°
FDG.Bebe	Bebe datos generales final
FDG.BebeNro	Si: N°
FDG.AcFis	Ac. Física datos generales final
FDG.AcFisNro	Si: N°
FDG.AcFisCual	Si:
	AUDIT questionnaire for the detection of alcohol consumption

Variable	Descripción
FETOH.FeNac	Fecha Nacimeinto
FETOH.FeEv	Fecha Evaluación ETOH final
FETOH.Edad	Edad Evaluación ETOH final
FETOH.Car	Carrera Evaluación ETOH final
FETOH.Sem	Semestre Evaluación ETOH final
FETOH.A1	(1) ¿Con qué frecuencia consume alguna bebida alcohólica?:
FETOH.A2	(2) ¿Cuántos TRAGOS de alcohol suele tomar en un día de consumo normal?:
FETOH.A3	(3) ¿Con que frecuencia toma 5 o más TRAGOS en un solo día?:
FETOH.A4	(4) En el curso del último año, ¿Con qué frecuencia ha sido incapaz de parar de beber una vez que había empezado?
FETOH.A5	(5) En el curso del último año, ¿Con qué frecuencia no pudo hacer lo que se esperaba de usted porque había bebido?
FETOH.A6	(6) En el curso del último año, ¿Con qué frecuencia ha necesitado beber en ayunas para recuperarse después de haber bebido mucho el día anterior?
FETOH.A7	(7) En el curso del último año, ¿Con qué frecuencia ha tenido remordimientos o sentimientos de culpa después de haber bebido?
FETOH.A8	(8) En el curso del último año, ¿Con qué frecuencia no ha podido recordar lo que sucedió la noche anterior porque había estado bebiendo?

Variable	Descripción
**FETOH.A9**	**(9) ¿Usted o alguna otra persona ha resultado herido porque usted había bebido?**
**FETOH.A10**	**(10) ¿Algún familiar, amigo, médico o profesional de la salud ha mostrado preocupación por su consumo de bebidas alcohólicas o le han sugerido que deje de beber?**
**FETOH.PT**	**Puntaje total ETOH**
	**SRQ Self Reporting Questionnaire**

Variable	Descripción
FSRQ.FeNac	Fecha Nacimiento
FSRQ.FeEv	Fecha Evaluación SRQ final
FSRQ.Edad	Edad Evaluación SRQ final
FSRQ.Car	Carrera Evaluación SRQ final
FSRQ.Sem	Semestre Evaluación SRQ final
FSRQ.1	1.�Tiene frecuentes dolores de cabeza?
FSRQ.2	2.�Tiene mal apetito?
FSRQ.3	3.�Duerme mal?
FSRQ.4	4.�Se asusta con facilidad?
FSRQ.5	5.�Sufre de temblor de manos?
FSRQ.6	6.�Se siente nervioso, tenso o aburrido?
FSRQ.7	7.�Sufre de mala digestión?
FSRQ.8	8.�Le cuesta pensar con claridad?
FSRQ.9	9.�Se siente triste?
FSRQ.10	10.�Llora con mucha frecuencia?
FSRQ.11	11.�Tiene dificultad para disfrutar sus actividades diarias?
FSRQ.12	12.�Tiene dificultad para tomar decisiones?
FSRQ.13	13.�Tiene dificultad para hacer su trabajo diario? (¿sufre usted con su trabajo?)
FSRQ.14	14.�Es incapaz de desempeñar un papel útil en si vida?
FSRQ.15	15.�Ha perdido interés en las cosas?
FSRQ.16	16.�Siente que usted es una persona inútil?
FSRQ.17	17.�Ha tenido la idea de acabar con su vida?
FSRQ.18	18.�Se siente cansado todo el tiempo?
FSRQ.19	19.�Tiene sensaciones desagradables en su estómago?
FSRQ.20	20.�Se cansa con facilidad?
FSRQ.PT	Puntaje total SRQ
	Laboratory evaluations

Variable	Descripción
BLAB.FeNac	Fecha Nacimiento
BLAB.FeEv	Fecha Evaluación basal
BLAB.Edad	Edad Evaluación basal
BLAB.Car	Carrera Evaluación basal
BLAB.Sem	Semestre Evaluación basal
BLAB.BDNF1	[BDNF] ng/ml basal
BLAB.BDNF2	[BDNF] ng/ml basal
BLAB.BDNF	[BDNF] ng/ml basal
BLAB.Met1	% Metilación basal
BLAB.Met2	% Metilación basal
BLAB.Met	% Metilación basal
FLAB.FeNac	Fecha Nacimiento
FLAB.FeEv	Fecha Evaluación final
FLAB.Edad	Edad Evaluación final
FLAB.Car	Carrera Evaluación final
FLAB.Sem	Semestre Evaluación final
FLAB.BDNF1	[BDNF] ng/ml final
FLAB.BDNF2	[BDNF] ng/ml final
FLAB.BDNF	[BDNF] ng/ml final
FLAB.Met1	% Metilación final
FLAB.Met2	% Metilación final
FLAB.Met	% Metilación final
BDNF.log.BF	log [BDNF] Basal/Final
MET.log.BF	log % Metilación Basal/Final
BDNF.RatBF	Ratio [BDNF] Basal/Final
MET.RatBF	Ratio % Metilación Basal/Final
SNP.BDNF	SNP BDNF

Description
Participant ID
Missing values
Sex: 0=F;1=M
Date of birth
Date of baseline Beck-II assessment
Age baseline Beck-II assessment
Career Beck-II baseline assessment
Semester baseline Beck-II assessment
Item 1: Sadness
Item 2: Pessimism
Item 3: Feelings of failure
Item 4: Loss of pleasure
Item 5: Feelings of guilt
Item 6: Feelings of Punishment
Item 7: Dissatisfaction with oneself
Item 8: Self-criticism
Item 9: Suicidal thoughts or desires
Item 10: Crying
Item 11: Agitation
Item 12: Loss of interest
Item 13: Indecision
Item 14: Uselessness
Item 15: Loss of Energy
Item 16: Change of Sleep Pattern
Item 17: Irritability
Item 18: Change in appetite
Item 19: Difficulty in Concentration
Item 20: Tiredness or fatigue
Item 21: Loss of interest in sex
Total Beck-II baseline score

Description
Date of birth
Date of final Beck-II assessment
Age final Beck-II assessment
Career final Beck-II assessment
Semester final Beck-II assessment
Item 1: Sadness
Item 2: Pessimism
Item 3: Feelings of failure
Item 4: Loss of pleasure
Item 5: Feelings of guilt
Item 6: Feelings of Punishment
Item 7: Dissatisfaction with oneself
Item 8: Self-criticism
Item 9: Suicidal thoughts or desires
Item 10: Crying
Item 11: Agitation
Item 12: Loss of interest
Item 13: Indecision
Item 14: Uselessness
Item 15: Loss of Energy
Item 16: Change of Sleep Pattern
Item 17: Irritability
Item 18: Change in appetite
Item 19: Difficulty Concentrating
Item 20: Tiredness or fatigue
Item 21: Loss of Interest in Sex
Beck-II final total score

Description
Date of birth
Date of birth Date of baseline SISCO-II assessment
Age baseline SISCO-II assessment
Career baseline SISCO-II assessment
Semester baseline SISCO-II assessment
1:Yes 0:No
Number
Overload of homework and academic work
The personality and character of teachers
Teachers' evaluations (exams, essays, research papers, etc.)
The type of work teachers ask you to do (map consultation, worksheets, essays, concept maps, etc.).
Failure to understand the topics covered in class.
Participation in class (answering questions, presentations, etc.)
Limited time to do the work
Group mates make faster progress on homework and/or academic work.
Sleep disturbances (insomnia or nightmares)
Chronic fatigue (permanent tiredness)
Headaches or migraines
Digestion problems, abdominal pain or diarrhoea
Scratching, nail biting, rubbing, etc.
Drowsiness or increased need for sleep.
Muscle aches and/or contractures
Skin reactions (rash, peeling, etc.)
Restlessness (inability to relax and be calm)
Anxiety, distress or despair
Increased or decreased food intake
Feelings of depression and sadness (downhearted)
Feelings of aggression or increased irritability
Sudden mood swings
Conflict or tendency to argue or quarrel
Isolation from others
Unwillingness to do your work as a student
Making a plan for the execution of your tasks
Self-praise
Venting and confiding (verbalising the situation of concern)
I tried to get something positive or beneficial out of the stressful situation.
Taking up a hobby (physical activity, reading, watching series, social networking, etc.)
Accompanying a loved one (family, pets, friends, etc.).
Total Stressors SISCO-II baseline assessment
Total Physical and Psychological Reactions SISCO-II baseline assessment
Total social behavioural reactions SISCO-II baseline assessment
Total Total Reaction Total SISCO-II baseline assessment
Total Academic Stress Measurement SISCO-II baseline assessment
Total Coping SISCO-II (Factor 4) baseline assessment
Total SISCO-II + coping baseline assessment

Description
Date of birth
Date of birth Date of final SISCO-II assessment
Age final SISCO-II assessment
Career final SISCO-II assessment
Semester Final SISCO-II assessment
1:Yes 0:No
Number
Overload of homework and academic work
The personality and character of teachers
Teachers' evaluations (exams, essays, research papers, etc.)
The type of work teachers ask you to do (map consultation, worksheets, essays, concept maps, etc.).
Failure to understand the topics covered in class.
Participation in class (answering questions, presentations, etc.)
Limited time to do the work
Group mates make faster progress on homework and/or academic work.
Sleep disturbances (insomnia or nightmares)
Chronic fatigue (permanent tiredness)
Headaches or migraines
Digestion problems, abdominal pain or diarrhoea
Scratching, nail biting, rubbing, etc.
Drowsiness or increased need for sleep.
Muscle aches and/or contractures
Skin reactions (rash, peeling, etc.)
Restlessness (inability to relax and be calm)
Anxiety, distress or despair
Increased or decreased food intake
Feelings of depression and sadness (downhearted)
Feelings of aggression or increased irritability
Sudden mood swings
Conflict or tendency to argue or quarrel
Isolation from others
Unwillingness to do your work as a student
Making a plan for the execution of your tasks
Self-praise
Venting and confiding (verbalising the situation of concern)
I tried to get something positive or beneficial out of the stressful situation.
Taking up a hobby (physical activity, reading, watching series, social networking, etc.)
Accompanying a loved one (family, pets, friends, etc.).
Total Stressors SISCO-II final assessment
Total physical and psychological reactions SISCO-II final assessment
Total social behavioural reactions SISCO-II final assessment
Total Total Reaction Total SISCO-II final assessment
Total Academic Stress Measurement SISCO-II final assessment
Total Coping SISCO-II (Factor 4) baseline assessment
Total SISCO-II + coping final assessment

Description

Description
Date of birth
Date Assessment date general baseline data
Age assessment general baseline data
Career general baseline data
Semester general baseline data
Psychiatric treatment general data baseline general data
Psychological treatment general data baseline
Drug treatment general data baseline
Yes: Which one(s)
Smoking general data baseline
If: No.
Drinks general data baseline
Yes: No
Physical activity general data baseline
Yes: No
Yes: No
Date of birth
Date of evaluation final general data
Age assessment final general data
Career final general data
Semester final general data
Psychiatric treatment final general data
Psychological treatment final general data
Drug treatment final general data
Yes: Which one(s)
Smoking final general data
If: No.
Drinks final general data
If: No.
Physical activity final general data
Yes: No
Yes: No

Description
Date of Birth
Date of Final ETOH Assessment
Age Final ETOH assessment
Career Final ETOH assessment
Semester Final ETOH assessment
(1) How often do you drink alcoholic beverages?
(2) How many DRINKS of alcohol do you usually drink in a normal drinking day?
(3) How often do you have 5 or more DRINKS in a single day?
(4) Over the course of the past year, how often have you been unable to stop drinking once you had started?
(5) In the past year, how often were you unable to do what was expected of you because you had been drinking?
(6) In the past year, how often have you needed to drink on an empty stomach to recover from heavy drinking the day before?
(7) In the past year, how often have you had regrets or feelings of guilt after drinking?
(8) In the past year, how often have you been unable to remember what happened the night before because you had been drinking?

Description
(9) Have you or anyone else been hurt because you had been drinking?
(10) Have any family members, friends, doctors or health professionals shown concern about your drinking or suggested that you stop drinking?
Total ETOH score

Description
Date of Birth
Date of Final SRQ Assessment
Age Final SRQ assessment
Career Final SRQ assessment
Semester Final SRQ assessment
1. Do you have frequent headaches?
2. Do you have a poor appetite?
3. Do you sleep badly?
4. Are you easily frightened?
5. Do you suffer from hand trembling?
6. Does he feel nervous, tense or bored?
7. Do you suffer from poor digestion?
8. Do you find it difficult to think clearly?
9. Do you feel sad?
10. Do you cry very often?
11. Do you have difficulty enjoying your daily activities?
12. Do you have difficulty making decisions?
13. Do you have difficulty in doing your daily work (do you suffer with your work?)?
14. Are you unable to play a useful role in your life?
15. Have you lost interest in things?
16. Do you feel that you are a useless person?
17. Have you ever had the idea of ending your life?
18. Do you feel tired all the time?
19. Do you have unpleasant feelings in your stomach?
20. Do you get tired easily?
Total SRQ score

Description
Date of birth
Date Baseline Assessment
Age Baseline assessment
Career Baseline assessment
Semester Baseline assessment
[BDNF] ng/ml baseline
[BDNF] ng/ml baseline
[BDNF] ng/ml baseline
% Basal methylation
% Basal methylation
% Basal methylation
Date of birth
Date Final assessment
Age Final assessment
Career Final assessment
Semester Final assessment
[BDNF] ng/ml final
[BDNF] ng/ml final
[BDNF] ng/ml final
Final % Methylation
Final % Methylation
Final % Methylation
log [BDNF] Baseline/Final
log % Methylation Basaline/Final
Ratio [BDNF] Baseline/Final
Ratio % Methylation Basaline/Final
BDNF SNP

Observaciones	Remarks
dd/mm/aaaa	dd/mm/yyyy
dd/mm/aaaa	dd/mm/yyyy
años	years

Observaciones	Remarks
dd/mm/aaaa	dd/mm/yyyy
dd/mm/aaaa	dd/mm/yyyy
años	years

Observaciones	Remarks
dd/mm/aaaa	dd/mm/yyyy
dd/mm/aaaa	dd/mm/yyyy
años	years

Observaciones	Remarks
dd/mm/aaaa	dd/mm/yyyy
dd/mm/aaaa	dd/mm/yyyy
años	years

Observaciones	Remarks

Observaciones	Remarks
dd/mm/aaaa	dd/mm/yyyy
dd/mm/aaaa	dd/mm/yyyy
Años	years
1:Sí 0:No	1:Yes 0:No
1:Sí 0:No	1:Yes 0:No
1:Sí 0:No	1:Yes 0:No
Nombre	Name
1:Sí 0:No	1:Yes 0:No
Número	Number
1:Sí 0:No	1:Yes 0:No
Número	Number
1:Sí 0:No	1:Yes 0:No
Número	Number
Cuál	Which
dd/mm/aaaa	dd/mm/yyyy
dd/mm/aaaa	dd/mm/yyyy
Años	years
1:Sí 0:No	1:Yes 0:No
1:Sí 0:No	1:Yes 0:No
1:Sí 0:No	1:Yes 0:No
Nombre	Name
1:Sí 0:No	1:Yes 0:No
Número	Number
1:Sí 0:No	1:Yes 0:No
Número	Number
1:Sí 0:No	1:Yes 0:No
Número	Number
Cuál	Which

Observaciones	Remarks
dd/mm/aaaa	dd/mm/yyyy
dd/mm/aaaa	dd/mm/yyyy
Años	years
0.Nunca<br>1.1 o menos al mes<br>2.De 2 a 4 veces al mes<br>3.De 2 a 3 veces a la semana<br>4.4 o más veces a la semana	0. never<br>1. 1 or less per month<br>2. 2 to 4 times a month<br>3. 2 to 3 times a week<br>4. 4 or more times a week.
0.1 ó 2<br>1.3 ó 4<br>2.5 ó 6<br>3.7, 8 ó 9<br>4.10 ó más	0. 1 or 2<br>1. 3 or 4<br>2. 5 or 6<br>3. 7, 8 or 9<br>4. 10 or more
0.Nunca<br>1.Menos de una vez al mes<br>2.Mensualmente<br>3.Semanalmente<br>4.A diario o casi a diario	0. never<br>1.Less than once a month<br>2. monthly<br>3. weekly<br>4.Daily or almost daily
0.Nunca<br>1.Menos de una vez al mes<br>2.Mensualmente<br>3.Semanalmente<br>4.A diario o casi a diario	0. never<br>1.Less than once a month<br>2. monthly<br>3. weekly<br>4.Daily or almost daily
0.Nunca<br>1.Menos de una vez al mes<br>2.Mensualmente<br>3.Semanalmente<br>4.A diario o casi a diario	0. never<br>1.Less than once a month<br>2. monthly<br>3. weekly<br>4.Daily or almost daily
0.Nunca<br>1.Menos de una vez al mes<br>2.Mensualmente<br>3.Semanalmente<br>4.A diario o casi a diario	0. never<br>1.Less than once a month<br>2. monthly<br>3. weekly<br>4.Daily or almost daily
0.Nunca<br>1.Menos de una vez al mes<br>2.Mensualmente<br>3.Semanalmente<br>4.A diario o casi a diario	0. never<br>1.Less than once a month<br>2. monthly<br>3. weekly<br>4.Daily or almost daily
0.Nunca<br>1.Menos de una vez al mes<br>2.Mensualmente<br>3.Semanalmente<br>4.A diario o casi a diario	0. never<br>1.Less than once a month<br>2. monthly<br>3. weekly<br>4.Daily or almost daily

Observaciones	Remarks
**0. No<br>1. Sí, pero no en el curso del último año<br>2. Sí, el último año**	**0. No<br>1. Yes, but not within the last year"<br>"2.<br>2. Yes, within the last year" "0.**
**0. No<br>1. Sí, pero no en el curso del último año<br>2. Sí, el último año**	**0. No<br>Yes, but not within the last year" "0.<br>Yes, in the last year" "0.**

Observaciones	Remarks
dd/mm/aaaa	dd/mm/yyyy
dd/mm/aaaa	dd/mm/yyyy
Edad	years
1:Sí 0:No	1:Yes 0:No
1:Sí 0:No	1:Yes 0:No
1:Sí 0:No	1:Yes 0:No
1:Sí 0:No	1:Yes 0:No
1:Sí 0:No	1:Yes 0:No
1:Sí 0:No	1:Yes 0:No
1:Sí 0:No	1:Yes 0:No
1:Sí 0:No	1:Yes 0:No
1:Sí 0:No	1:Yes 0:No
1:Sí 0:No	1:Yes 0:No
1:Sí 0:No	1:Yes 0:No
1:Sí 0:No	1:Yes 0:No
1:Sí 0:No	1:Yes 0:No
1:Sí 0:No	1:Yes 0:No
1:Sí 0:No	1:Yes 0:No
1:Sí 0:No	1:Yes 0:No
1:Sí 0:No	1:Yes 0:No
1:Sí 0:No	1:Yes 0:No
1:Sí 0:No	1:Yes 0:No
1:Sí 0:No	1:Yes 0:No
Puntaje de corte:11	Cut-off score:11

Observaciones	Remarks
dd/mm/aaaa	dd/mm/yyyy
dd/mm/aaaa	dd/mm/yyyy
Años	years
Medición 1	Measurement 1
Medición 2	Measurement 2
Promedio mediciones	Average measurement
Medición 1	Measurement 1
Medición 2	Measurement 2
Promedio mediciones	Average measurement
dd/mm/aaaa	dd/mm/yyyy
dd/mm/aaaa	dd/mm/yyyy
Años	years
Medición 1	Measurement 1
Medición 2	Measurement 2
Promedio mediciones	Average measurement
Medición 1	Measurement 1
Medición 2	Measurement 2
Promedio mediciones	Average measurements
Promedio mediciones	Average measurements
Promedio mediciones	Average measurements
promedio mediciones	average measurements
promedio mediciones	average measurements
0=Val/Val 1=Val/Met 2=Met/met	0=Val/Val 1=Val/Met 2=Met/met
