# Supplementary material for: Increased academic stress is associated with decreased plasma BDNF in Chilean college students
Source: PeerJ. 2023 Nov 3;11:e16357. doi: 10.7717/peerj.16357 (PMC10629390; doi:10.7717/peerj.16357)
Supplement: Supplemental Information 3 — Some sections of this paper were written with the help of the GPT-4 AI model. A document detailing the Procedure used for the improvement of writing in English is available as supplementary material. However, the results of this study are presented clearly, honestly, and without fabrication, falsification, or inappropriate data manipulation. [file peerj-11-16357-s003.pdf]

Procedure used for the improvement of writing in English.

1. Writing concepts considered: clarity, coherence, formality, grammar, and orthography.
2. Model of IA used: Chat GPT-4
3. Promt used:
    a. Forget all of the above. I want you to act like a great academic, specialized in writing scientific articles for prestigious scientific and academic journals of high impact. In this context you will be asked to improve the writing of a text. You will do this to the best possible standard, considering a maximum length of 16 words per sentence. Since you are a great academic, you will be learning and will always deliver the best writing. You will only improve the writing without adding any concept, idea, information, analysis, or conclusion coming from you, as you are not being asked to do so. Each time you are asked to improve the wording of a given text you will receive the instruction "IW:" followed by the text whose wording you are asked to improve to the best level. Remember that the maximum length is 16 words per sentence, you will be limited only to improve the wording without adding concepts, ideas, information, analysis, or conclusion. Concepts that you will not be able to change: Stressors, Physical and psychological reactions, Social behavioural reactions, Coping. understood? shall we proceed?
    b. IW: “text to improve writing”
4. Final proofreading: writewrise.io platform (https://web.writewise.io/), with institutional access.
